# Dogs demonstrate the existence of an epileptic seizure odour in humans

**DOI:** 10.1038/s41598-019-40721-4

**Published:** 2019-03-28

**Authors:** Amélie Catala, Marine Grandgeorge, Jean-Luc Schaff, Hugo Cousillas, Martine Hausberger, Jennifer Cattet

**Affiliations:** 1 Univ Rennes, Normandie Univ, CNRS, EthoS (Éthologie animale et humaine) - UMR 6552, F-35380 Paimpont, France; 2 Association Handi’Chiens, 13 Rue de l’Abbé Groult, Paris, France; 3 Centre d’Observation et de Cure pour Enfants Epileptiques, Ets OHS de Lorraine, 46 rue du doyen J. Parisot, Flavigny-sur-Moselle, France; 4 Service de Neurologie du CHRU de Nancy, 29, avenue du Maréchal de Lattre de Tassigny, Nancy, France; 5 Univ Rennes, Normandie Univ, CNRS, EthoS (Éthologie animale et humaine) - UMR 6552, F-35000 Rennes, France; 6 CNRS, Univ Rennes, Normandie Univ, EthoS (Éthologie animale et humaine) - UMR 6552, F-35380 Paimpont, France; 7 Medical Mutts, Indianapolis, Indiana, USA

## Abstract

Although different studies have shown that diseases such as breast or lung cancer are associated with specific bodily odours, no study has yet tested the possibility that epileptic seizures may be reflected in an olfactory profile, probably because there is a large variety of seizure types. The question is whether a “seizure-odour”, that would be transversal to individuals and types of seizures, exists. This would be a pre requisite for potential anticipation, either by electronic systems (e.g., e-noses) or trained dogs. The aim of the present study therefore was to test whether trained dogs, as demonstrated for cancer or diabetes, may discriminate a general epileptic seizure odor (different from body odours of the same person in other contexts and common to different persons). The results were very clear: all dogs discriminated the seizure odour. The sensitivity and specificity obtained were amongst the highest shown up to now for discrimination of diseases. This constitutes a first proof that, despite the variety of seizures and individual odours, seizures are associated with olfactory characteristics. These results open a large field of research on the odour signature of seizures. Further studies will aim to look at potential applications in terms of anticipation of seizures.

## Introduction

Changes in human odour, as symptoms of specific diseases, have been noted since Hippocrates’ time. Only recently though has human body odour been proven to vary in relation with health disorders. For example, the composition of exhaled breath is different in patients with lung cancer, inflammatory lung or liver disease, hepatic or renal dysfunction or diabetes^1–3^.

Identification of odour profiles has become an important issue and some disease-specific volatile organic compounds (VOCs) have been found. Detection of these particular odours at the early stages of diseases is crucial and new screening technologies (e.g. electronic nose^4^) have been developed. However, the existing technologies are still not sufficient. Thus, in their review, Szulejko *et al*.^5^ pointed out the difficulties, such as individual variations due to many factors and the fact that patients will never exhale clean air. Moreover, the large signal/noise ratio recurrent with trace analysis and database analysis also makes the isolation of the specific compounds difficult.

Because of their known high olfactory abilities, dogs have been used to detect breast or lung cancer, diabetes or kidney diseases with some success^6,7^, although results are sometimes contradictory, perhaps because of the variety of training procedures (e.g. urine, sweat or breath odours)^8–10^. Nevertheless, dogs appear to outperform electronic noses^11^, as they require an average VOC concentration of less than 0.001 part per billion (1 × 10–12)^12^ whereas e-noses have a detection threshold of 100 to 400 per billion (1 × 10–7)^5^.

In colorectal cancer, the most noninvasive test is the fecal occult blood test, where sensitivity reaches 43.8% for colorectal neoplasia^13^, whereas canine scent sensitivity for breath samples was 91%. This means that dogs can provide a more successful and non-invasive screening method^8^. Also called medical detection, the detection of temporary physiological changes such as glycemic changes^14–16^ or migraines^17^ has been demonstrated in trained dogs, enabling the patient to anticipate oncoming crises within minutes to hours before.

A similar anticipation would be very important for epileptic patients, who could then seek a more secure environment before a seizure. Epilepsy is a neurologic disorder characterized by recurrent seizures^18^ that can be classified in many types and imply various symptoms^19^. Epilepsy may arise from genetic or structural modifications in the brain, brain infections, head injuries, strokes or tumors^18^. Also various comorbid psychiatric disorders are commonly associated with epilepsy, such as anxiety and depression^20–23^. This can explain why epilepsy is individual-specific. This high variability may explain why no study has been undertaken on a potential seizure-specific odour yet. Anecdotal reports suggest that some pet dogs alert their epileptic owners about an imminent seizure, but the cues potentially used by these dogs are unknown^24^. Some associations/institutions have undertaken to train dogs to detect bodily odours, but on an individual (future owner)-specific basis (breath, sweat)(J. Cattet, pers. obs.) and with no clear proof of concept yet.

In the present study, we hypothesized that there may be a seizure-specific olfactory component that would be common to different individuals and types of seizures.

We tested this hypothesis by using a validated protocol^15^ which consisted of presenting to trained dogs complex odours (breath and sweat) obtained from each epileptic patient during a seizure, outside a seizure (calm activity) or during sports sessions (to test for a potential sweat specificity).

We could thus test: (1) whether dogs are able to discriminate a seizure sample amongst odours obtained in other contexts, (2) whether this was not a mere discrimination of sweat, (3) whether dogs could detect a seizure odour from different patients, which would imply that there are volatile elements that are common to different types or causes of epilepsy. As we focused on ictal odour and not on pre-ictal, we did not make assumptions on seizure-alerting abilities of dogs, or on the timing of such anticipation, in this study.

Five neutered dogs (three females, two males of various breeds; aged 2 to 5 years, mean: X = 2.9 years, see Supplementary Table S2) from Medical Mutts (Indianapolis, USA), trained to respond in a particular way (approaching and standing above the can containing the target odour, i.e. “response behaviour”) to bodily odours of patients with different diseases or disorders (diabetes, anxiety or epilepsy) were individually tested on a seven-choice task. At each trial, seven scent samples from one patient were presented in opaque cans: one from a seizure, two from a sports session, four taken pseudo-randomly on different days during calm activity. The samples were from 5 patients with different types of epilepsy (mostly partial symptomatic from various causes: frontal lobe complex partial seizure (n = 2), temporal lobe complex partial seizure (n = 2), continuous simple partial seizures and temporal lobe complex partial seizure (n = 1) due to Rasmussen syndrome (n = 1), Ring Chromosome 20 (n = 1), and cerebral malformations (dysplasia) (n = 3)). As the odours used for training were not from the patients, the dogs included in the present study were completely naïve to these particular odours. Each test trial began when the dog entered the room, ended with the response behaviour, and lasted a maximum of 5 minutes, with a 2-min-break between each trial in order to remove and replace the cans while the dog waited in another room. For all dogs, the trials were repeated five times for the odours from one person (patient A). Then, after a two-hour break, each dog was presented randomly the four remaining sets of scents from different persons (patients B, C, D, and E). Thus, each dog was involved in 9 trials in total.

## Results

All dogs succeeded in each trial, exhibiting the “response behaviour” on the correct can (i.e. with seizure odour) in much less than the criterion of 5 min (mean latency ± SD: 9.3 ± 2.08 sec). Since in each trial, only one out of the 7 cans contained the seizure odour, the dogs’ performance was well above the chance sensitivity (proportion of actual “positives” that were correctly identified^15^) level of 14%, with 67% to 100% sensitivity. Specificity (proportion of actual “negatives” (i.e. no-response behaviour on a non-epilepsy sample) also ranged from 95% to 100%. Three of the 5 dogs performed at 100% sensitivity and specificity. The two remaining dogs displayed 67% sensitivity and 95% specificity, thus quite high performances still (Table 1, and Supplementary Table S1).

**Table 1:** Latency, sensitivity and specificity for 5 dogs evaluated for representative results.

Dogs	Sensitivity (%)	Specificity (%)	Binomial Test P Value
Casey	100	100	<1E^−5^
Dodger	100	100	<1E^−5^
Lana	67	95	4E^−4^
Zoey	100	100	<1E^−5^
Roo	67	95	4E^−4^
Total	86,8	98	—

Calculation of p value assumed binomial distribution probability of success = 0.14 and trials = 9 per dog. Total samples presented to each dog = 63.

**Figure 1:**
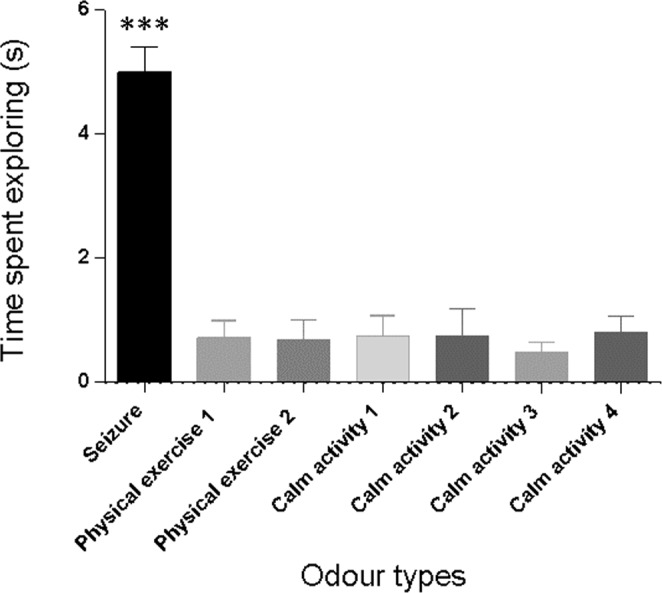
Mean time spent exploring (seconds) each odour type and 95% confidence intervals represented. (***p < 0.001). Odour types were from a seizure (*seizure*), two from a sports session (*physical exercise 1 & 2*), and four taken pseudo-randomly on different days during calm activity (*calm activity 1, 2, 3 & 4*).

The dogs did not spend the same amount of time exploring the different samples (X² = 117.1, p < 0.0001). They examined for a much longer time the seizure samples (mean, 5.01 ± 1.34 sec) compared to the other samples (pooled data, mean, 0.70 ± 0.11 sec) (post hoc multiple comparison test, p < 0.0001; Fig. 1).

When comparing the responses of the dogs to the odours of the different patients, it appeared that the results were concordant (Kendall’s concordance test permutational probabilities based upon 999 random permutations: W = 0.448, F = 3.246, prob F = 0.021, Chi2 = 13.441, Prob. Perm = 0.020).

**Figure 2:**
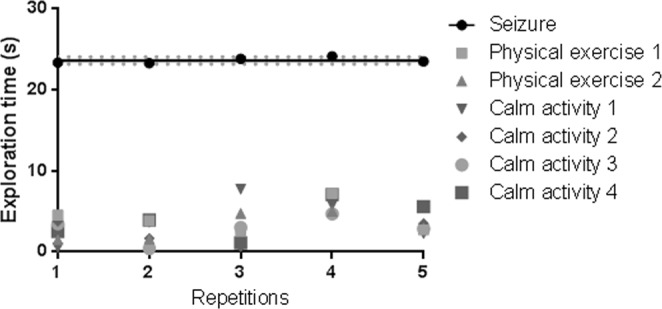
Exploration time (all dogs and five repeated subjects pooled) per type of odour, per repetition. Line = regression linear line for seizure sample, dots = means of exploration times per sample type, dotted lines = standard error for seizure sample. Prism’s linear regression analysis revealed no effect of learning (slope not different from 0) in any of the conditions [seizure: *F*(1,3) = 1.037, *p* = 0.38; Physical exercise 1: *F*(1,3) = 0.001, *p* = 0.97, Physical exercise 2: *F*(1,3) = 1.718, *p* = 0.28; d 1: *F*(1,3) = 0.32, *p* = 0.607; d 2: *F*(1,3) = 0.318, *p* = 0.612; d 3: *F*(1,3) = 0.329, *p* = 0.606; d 4: *F*(1,3) = 1.853, *p* = 0.267.

The dogs showed the same interest in the seizure odour through all five repetitions with the same individual’s (patient A) odour, and from the very beginning (Fig. 2). The very clear discrimination exhibited therefore could not just result from learning from these samples. Also, all of the dogs choose correctly in the first trial and the number of false positives (more time spent on “incorrect” samples) did not decrease across repetitions.

## Discussion

This study, in which trained dogs were confronted with bodily odours from epileptic patients sampled during and outside seizures, shows that these dogs were clearly able to discriminate the seizure odours from odours of the same patient outside seizures and for all patients tested. From the first trial on, they responded to the “right” odour and explored it longer than any of the other odours. This clearly demonstrates for the first time that there is indeed a seizure-specific odour across individuals and types of seizures.

This is especially remarkable as these dogs were trained for scent discrimination of various diseases or conditions. Future studies should involve a larger number of dogs and patients, but the dog sample size was in accordance with those in most studies on medical scent detection^25–29^, as trained dogs require a long preparation. The patient sample size was limited for practical and ethical reasons relative to epilepsy specificities and would gain to be increased in the future. However, the results are extremely clear and constitute a first step towards identifying a seizure-specific odour

This demonstration that dogs can discriminate a seizure-specific odour does indeed open new lines of research on the potential general odour profile involved. Chemical analyses such as gas chromatography or mass spectrometry would have to be used to identify this profile beyond individual and seizure type variations. It could lead to significant improvements in terms of seizure detection or prediction systems. The responses observed here show that generalization is much higher than expected. Moreover, nothing is known about the potential pre-ictal presence of an odour, which would be important to help patients anticipate and seek security before a seizure occurs.

One interesting aspect is also that in the case of epileptic seizures, dogs have to detect a temporary odour contrary to the detection of chronic states with potentially a permanent similar odour (e.g. cancer^8,28^). Nevertheless, the sensitivity and selectivity observed here were amongst the highest shown up to now for the olfactory discrimination of diseases by dogs (13^30^ to 100%^29^ in cancer detection, 50^14,15^ to 87.5%^15^ sensitivity in diabetes). This may be related to the mix of odours used, which included breath and skin odour, and which have been rather successfully used^15,31,32^.

Nevertheless, in real-life conditions, diabetes dogs appeared to show a poor reliability: 26.2 to 59.2% in sensitivity^33,34^; 36% sensitivity and the PPV of 12% only due to inappropriate alerts^35^ with a large variability across dogs.

Inconsistencies in the field of canine biomedical detection both within and across studies are likely attributable to several factors, including differences in dog characteristics (e.g. breed, age, sex), differences in training protocols and training time, experimental setup, and sample collection and storage (reviewed by^9,36^).

An intriguing issue is the reports of pet dogs that, without any formal training, alert for a seizure^37–39^. There is no clear knowledge of “the success rate” of these dogs nor on what cues they use. Beyond olfaction, responses to subtle visual cues may also be involved. The dogs may have learned to associate the particular behaviour of their owners with the specific odour or postural alterations that may precede the seizure. Only further studies could help answer these questions. The first aim of the study was to demonstrate that a specific odour exists and dogs are a good medium for detecting them. Further studies will aim at determining further the temporal dynamics of odour changes and dogs’ responses.

In any case, we believe that these first results make an important contribution to the field of epileptic research and reopens the possibility that seizures may be anticipated by looking further at olfactory characteristics. This possibility was previously set aside because of the belief that epilepsy and seizure types were too individual-specific for a general cue to be found. Dogs demonstrate that there is hope in this direction.

## Material and Methods

### Ethics

The study received approval by the institutional review board of OHS de Lorraine for collection techniques used to obtain odour samples from patients with epilepsy. All institutional (Medical Mutts) and published guidelines (Assistance Dogs International) for the use of positive reinforcement in the care and use of the dogs were followed. The Animal Ethics Committee of Rennes was consulted for this study and confirmed there was no need to request an authorization for this research, since it did not affect dog welfare.

The present research was non-invasive and did not involve pharmacological interventions. Hence, in accordance with the Ethics Committee’s guidelines, parents gave only an informed written consent to allow the child’s participation in the experiment prior to their inclusion in the study.

### Subjects

Five trained dogs participated in this study (Supplementary Table S2). All subjects lived in Medical Mutts facilities (USA) and had never been exposed to the experimental five patient odours. Medical Mutts selected dogs from shelters to be trained by professional trainers as service dogs. Selection criteria included temperament traits compatible for service dog training^40^ and physical aptitudes. Dogs are trained for obedience for certification for public access in addition to scent discrimination. Dogs spent one hour per day, five days per week in a training session^15^.

### Odour samples

Patients were recruited in the Healthcare Centre OHS Flavigny, Flavigny sur Moselle, France. Ten participants were initially invited to participate after medical recommendations. One declined and the other 4 did not experience a seizure through the sample collection time of one week in December 2017. Thus, odours were collected from 5 females who were residents of the Healthcare Centre OHS. All had a confirmed diagnosis of epilepsy (See Supplementary Table S3) and none experienced psychogenic non-epileptic seizures. Psychogenic non-epileptic seizures (exclusive or in addition to epileptic seizures) were actually a factor of exclusion when recruiting patients on the basis of their medical diagnosis. Since they all lived in a medicalized structure at the time of the sampling, the diagnosis was further confirmed.

They received the same food without dietary restrictions. Since the seizures could be experienced at any time, the collection of epilepsy seizure samples could occur randomly at any time (night or day), whereas all other samples were collected during day. Patients were not given any specific recommendation (e.g. regarding hormonal contraception, perfume, smoking etc.), so as to maintain an “ecological” individual variability.

### Odour sampling procedure

Three types of samples were collected: (a) a “seizure sample”, collected during or right after (<5 min) an ictal event; (b) a “physical exercise sample”, collected right after (<5 min) moderate exercise (defined as 1 min during which the patient ran approximately 30 m, and did leg and arm movements as step-touch and punches). Physical exercise samples served as control for movements or arousal that can be made or experienced during a seizure^41,42^. And (c) a “day sample” was collected at a pseudo-random moment during calm activity (e.g. drawing, paper crafting) in day time. The non-seizure samples were collected a minimum of 6 hours before or after a seizure in order to avoid a pre- or post-ictal collection.

For each patient, two physical exercise samples were collected on two different days and four day samples on four different days.

Odour collection was performed in accordance with the procedure used at Medical Mutts for training assistance dogs^15^: Patients were instructed to use a sterile cotton pad (10*10 cm, 4 fold) to wipe their hands, forehead and back of the neck, allowing a multiplicity of odour origins. The cotton pad was then placed into a zip-locked bag (Ziplock brand, SC Johnson, Racine, WI, USA) and the patient was instructed to exhale into the bag before sealing it. Two cotton pads were collected from each patient, for each of the 7 types of odour. Each bag was labelled with the patient’s initials, day and time of collection and the corresponding odour type (normal, physical exercise or seizure). The samples were stored in the freezer (Temperature −12 °C) then shipped from France to USA at ambient temperature and stored again in the freezer until use (average of 47 days, SD = 18). Freezing has been shown to preserve samples of human body odour without altering perceptions of these odours and freeze–thaw cycles to not affect sample quality^43^.

### Training

The dogs that participated in the study were trained using positive reinforcement methods [12, 13]. They underwent the general training for assistance dog (30 commands, staying under control in public areas, etc.).

More specifically, the training for SAD required three main steps. First a positive association is created between the scent of a seizure and treats in order to induce a conditioned emotional response. Then the dog learns to go to the scent and to stand still above it. This indication behaviour (dog standing above the scent) is developed by adding distance and duration and using the command “Check”. The second step consisted in a discrimination task, for additional scents (distractors, inter-ictal scent) were progressively added and the dog learned to only indicate the seizure scent.

In the third phase of training, the seizure scent was placed on a person. The ‘stay-stand’ indication was replaced by teaching the dog to alert by a poke of the nose on the person’s body.

The odours used for training were not from the patients. Therefore, the dogs included in the present study were completely unfamiliar with these particuliar odours.

### Experimental Setup

Tests took place from January 2018 to February 2018, in a closed room (9.1 × 6.1 m, 4 m high, 21 °C in average) (Supplementary Fig. S1) in the training facility. No experimenter was present in the testing room during a trial (see also^15^). Experimenter was in a room nearby, separated from the first one by a wooden door. One camera was linked to a monitoring system, and another recorded the trials. At the beginning of a trial, the room was open, the trainer gave the command (“Check”), then closed the door and stayed out of view or reach in the second room. The dog was free to explore the experimental set-up until the correct identification of the seizure sample. It was then rewarded with a treat from an automatic dispenser remotely activated by the trainer (Pet Tutor®). Then the trainer opened the door and called the dog out of the room. The dog stayed in the second room in between two trials, while the experimenter changed the samples and put the cans in the right locations for the next trial.

The experimental set-up consisted of seven steel cans (18 cm high × 17 cm in diameter), each containing one small canister (5.2 cm in diameter, 2 cm high) in which one odour sample was placed. These canisters prevented the dog from being in direct contact with samples. The cans were in a semicircle arrangement, equidistant (2.08 m) from the Pet Tutor®. Each can contained an odour sample that could be either a seizure, physical exercise or calm activity sample. Compared to the literature on similar experiments, this is a high average for the number of samples presented in line-up methods and the distance between cans (about 50 centimeters) was comparable^8,15,26^.

At each test, all seven cans contained an odour from the same person: one seizure sample, two physical exercise samples and four calm sample.

### Test procedure

Trials took place as follows: each dog had the first 5 trials using the same person’s odour, a 2-hour break, and then one trial with each of the 4 remaining patients (See Fig. 2). Thus, each dog had 9 trials in one day. Each test trial lasted a maximum of 5 minutes, with a 2 min break between each trial in order to remove and replace the cans. Odour locations were pseudo-randomly determined prior to the sessions and changed between trials, with the constraint that the same location could not be used more than three times in a row for the same type of odour in order to avoid learning bias and inducing A-not-B errors (Smith *et al*., 1999).

### Behavioural coding

Each trial was videotaped (30 fps, Sony Handycam HDR-CX380) and coded using frame-by-frame playback for accuracy of determination of investigatory behaviour. The latency of first response was measured as well as the time spent by the dog in the olfactory investigation of cans (i.e. nose at less than ten centimeters from the cans^44–46^). Given the indication behavior (remain standing with the head above the cans), an indication was counted if it lasted more than 3 seconds. The same person (A.C.), blind to the cans’ contents, coded all trials. Another coder, unfamiliar with the goals of the study, verified the reliability of this coding on all trials. Inter-observer agreement was high (*κ* = 0.91).

### Statistical analyses

Sensitivity and specificity were calculated after pooling the data across all trials for all dogs in accordance with^15^.

In addition, as the data were not normally distributed, non-parametric statistical tests were used^47^. Performances for individual dogs were statistically assessed with binomial tests. A Friedman test compared the time spent exploring the different sample categories, with all dogs and trials pooled. To assess potential differences between the odours of each patient, all dogs were pooled on the cumulated exploration time for the five different patients (first presentation of the repeated person and the last four persons). The concordance among dogs’ performances according to persons was estimated using a Kendall test by permutation.

## Supplementary information

Supplementary Information

## Data Availability

Raw measurement data of subjects and videos are available upon request.
