# Supplementary material for: Dogs demonstrate the existence of an epileptic seizure odour in humans
Source: Sci Rep. 2019 Mar 28;9:4103. doi: 10.1038/s41598-019-40721-4 (PMC6438971; doi:10.1038/s41598-019-40721-4)

**Supplementary Information:**

**Dogs demonstrate the existence of an epileptic seizure odour in humans**

Amélie Catala, Marine Grandgeorge, Jean-Luc Schaff, Hugo Cousillas, Martine Hausberger, Jennifer Cattet

**Table S1**: Dogs performances. Latency, sensitivity and specificity for 5 dogs evaluated for representative results. Calculation of p value assumed binomial distribution probability of success = 0.14 and trials = 9 per dog. Total samples presented to each dog = 63. FN : false negative, FP false positive, TN true negative, TP true positive.

| Dogs | Latency in sec (mean±SD) | Total samples | N (FN) | N (FP) | N (TN) | N (TP) | Sensitivity (%) | Specificity | Binomial test p value |
| --- | --- | --- | --- | --- | --- | --- | --- | --- | --- |
| Casey | 8.2 ±2.4 | 63 | 0 | 0 | 54 | 9 | 100 | 100 | <1E^-5^ |
| Dodger | 7.5 ±2.4 | 63 | 0 | 0 | 54 | 9 | 100 | 100 | <1E^-5^ |
| Lana | 12.8 ±4.1 | 63 | 3 | 3 | 51 | 6 | 67 | 95 | 4E^-4^ |
| Zoey | 9.1 ±4.0 | 63 | 0 | 0 | 54 | 9 | 100 | 100 | <1E^-5^ |
| Roo | 8.6 ±5.2 | 63 | 3 | 3 | 51 | 6 | 67 | 95 | 4E^-4^ |
| Total |  | 315 | 6 | 6 | 276 | 39 | 86,8 | 98 | ---- |

**Table S2**: Name, sex, age, breed and training information of dogs.

| DOGS | Sex | Age (years) | Breed | Sessions | Repetitions |
| --- | --- | --- | --- | --- | --- |
| Casey | F | 2 | Golden - Labrador retriever mix | 37 | 786 |
| Dodger | M | 2 | Labrador retriever mix | 28 | 585 |
| Lana | F | 2 | Border collie mix | - | - |
| Zoey | F | 3,5 | Chesapeake bay retriever mix | 30 | 452 |
| Roo | M | 5 | Dachshund - Australian shepherd mix | - | - |

A session implied olfaction task whatever sample type (anxiety, diabetes or epilepsy), a repetition was counted each time the dog was rewarded.

**Table S3**: Patients informations

| Patient | Sexe | Age (years) | Cause of epilepsy | Epilepsy type |
| --- | --- | --- | --- | --- |
| A | Female | 17 | Rasmussen syndrome | Continuous simple partial seizures and temporal lobe complex partial seizure |
| B | Female | 14 | Cerebral malformations (dysplasia) | Frontal lobe complex partial seizure |
| C | Female | 15 | Cerebral malformations (dysplasia) | Frontal lobe complex partial seizure |
| D | Female | 17 | Cerebral malformations (nodular heterotopia) | Temporal lobe complex partial seizure |
| E | Female | 11 | Ring Chromosome 20 | Temporal lobe complex partial seizure |

**Figure S1:** Room layout for testing SADs


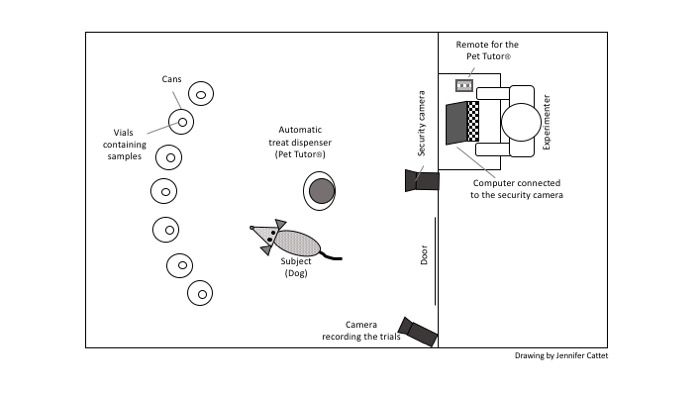

Supplement: Supplementary file 1 — Supplementary Information [file 41598_2019_40721_MOESM1_ESM.docx]
